# Deep Learning-Based Estimation of Axial Length and Subfoveal Choroidal Thickness From Color Fundus Photographs

**DOI:** 10.3389/fcell.2021.653692

**Published:** 2021-04-09

**Authors:** Li Dong, Xin Yue Hu, Yan Ni Yan, Qi Zhang, Nan Zhou, Lei Shao, Ya Xing Wang, Jie Xu, Yin Jun Lan, Yang Li, Jian Hao Xiong, Cong Xin Liu, Zong Yuan Ge, Jost. B. Jonas, Wen Bin Wei

**Affiliations:** ^1^Beijing Key Laboratory of Intraocular Tumor Diagnosis and Treatment, Beijing Ophthalmology and Visual Sciences Key Laboratory, Medical Artificial Intelligence Research and Verification Key Laboratory of the Ministry of Industry and Information Technology, Beijing Tongren Eye Center, Beijing Tongren Hospital, Capital Medical University, Beijing, China; ^2^Beijing Eaglevision Technology Co., Ltd., Beijing, China; ^3^Beijing Ophthalmology and Visual Science Key Laboratory, Beijing Tongren Eye Center, Beijing Tongren Hospital, Beijing Institute of Ophthalmology, Capital Medical University, Beijing, China; ^4^eResearch centre, Monash University, Melbourne, VIC, Australia; ^5^ECSE, Faculty of Engineering, Monash University, Melbourne, VIC, Australia; ^6^Department of Ophthalmology, Medical Faculty Mannheim, Heidelberg University, Mannheim, Germany

**Keywords:** deep learning, convolution neural network, axial length, subfoveal choroidal thickness, fundus photography, fundus image

## Abstract

This study aimed to develop an automated computer-based algorithm to estimate axial length and subfoveal choroidal thickness (SFCT) based on color fundus photographs. In the population-based Beijing Eye Study 2011, we took fundus photographs and measured SFCT by optical coherence tomography (OCT) and axial length by optical low-coherence reflectometry. Using 6394 color fundus images taken from 3468 participants, we trained and evaluated a deep-learning-based algorithm for estimation of axial length and SFCT. The algorithm had a mean absolute error (MAE) for estimating axial length and SFCT of 0.56 mm [95% confidence interval (CI): 0.53,0.61] and 49.20 μm (95% CI: 45.83,52.54), respectively. Estimated values and measured data showed coefficients of determination of *r*^2^ = 0.59 (95% CI: 0.50,0.65) for axial length and *r*^2^ = 0.62 (95% CI: 0.57,0.67) for SFCT. Bland–Altman plots revealed a mean difference in axial length and SFCT of −0.16 mm (95% CI: −1.60,1.27 mm) and of −4.40 μm (95% CI, −131.8,122.9 μm), respectively. For the estimation of axial length, heat map analysis showed that signals predominantly from overall of the macular region, the foveal region, and the extrafoveal region were used in the eyes with an axial length of < 22 mm, 22–26 mm, and > 26 mm, respectively. For the estimation of SFCT, the convolutional neural network (CNN) used mostly the central part of the macular region, the fovea or perifovea, independently of the SFCT. Our study shows that deep-learning-based algorithms may be helpful in estimating axial length and SFCT based on conventional color fundus images. They may be a further step in the semiautomatic assessment of the eye.

## Introduction

Axial length and subfoveal choroidal thickness (SFCT) belong to the most important biometric parameters of the eye and are directly or indirectly associated with axial ametropias and maculopathies such as myopic macular degeneration and pachychoroid-associated macular diseases, to name only a few (Fujiwara et al., 2009; Spaide, 2009; Saka et al., 2010; Cheung et al., 2013; Shao et al., 2014; Ohno-Matsui et al., 2015; Tideman et al., 2016; Yan et al., 2018a, b; Lim et al., 2020; Peng et al., 2020). Although both parameters can relatively easily and non-invasively be determined with relative high precision, their measurements necessitate costly ophthalmological devices and equipment, which are not readily available and the use of which are personal dependent and time consuming. Incentives have, therefore, started to assess axial length and SFCT by other means than the conventional measurement devices. Since fundus photographs can be taken with easily available devices including smartphones (Bastawrous et al., 2016; Toy et al., 2016; Muiesan et al., 2017; Mamtora et al., 2018), we conducted this study to assess whether readily taken photographs of the ocular fundus could serve for an estimation of both biometric parameters with the application of deep-learning-based algorithms. In previous studies, artificial intelligence has already been shown to be helpful in the assessment of medical images and diagnosis of diseases (Ting et al., 2017; Biousse et al., 2020; Milea et al., 2020). Deep learning, known as a subset of artificial intelligence, allows computational systems to learn representations directly from a large number of images without designing explicit hand-crafted features (LeCun et al., 2015). The applications of deep-learning techniques trained on color fundus images have produced systems with competitive or close-to-expert performance for an automatic detection of ophthalmic diseases, including diabetic retinopathy (Cao et al., 2020; Gargeya and Leng, 2017; Ting et al., 2017), age-related macular degeneration (Burlina et al., 2017; Grassmann et al., 2018; González-Gonzalo et al., 2020), retinopathy of prematurity (Wang et al., 2018; Mao et al., 2020), glaucoma (Hemelings et al., 2020), and other disorders (Shah et al., 2020); assessment of ocular and systemic risk factors such as age, gender, body mass index, and blood pressure; estimation of the refractive error (Poplin et al., 2018; Varadarajan et al., 2018; Chun et al., 2020).

## Materials and Methods

**FIGURE 1:**
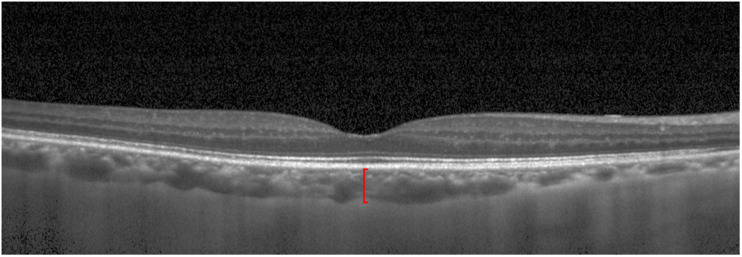
Optical coherence tomographic image (enhanced depth imaging mode) showing the retina and the choroid. Red line: subfoveal choroidal thickness.

The Beijing Eye Study 2011 was a population-based, cross-sectional study conducted in Northern China (Wei et al., 2013; Yan et al., 2015). The Medical Ethics Committee of the Beijing Tongren Hospital approved the study protocol, and all participants gave an informed consent. The study was carried out in five communities in the urban area of Haidian district and three communities in the village area of Daxing District. The only eligibility criterion for inclusion in the study was an age group of ≥ 50 years. In total, 3468 individuals (1963 female, 56.6%) participated in the eye examination. Optical low-coherence reflectometry (Lensstar 900 Optical Biometer, Haag-Streit, 3098 Koeniz, Switzerland) was used for biometry of the right eyes for the measurement of axial length. After medical mydriasis, photographs of the macula and optic disk were taken using a 45° fundus camera (Type CR6-45NM, Canon Inc, Lake Success, NY, United States). The SFCT was measured using spectral-domain optical coherence tomography (SD-OCT) (Spectralis, wavelength of 870 nm; Heidelberg Engineering Co, Heidelberg, Germany) applying the enhanced depth imaging (EDI) modality. Seven OCT sections, each comprising 100 averaged scans, were obtained in a rectangle measuring 5° × 30°, centered onto the fovea. The horizontal section running through the center of the fovea was selected for further analysis. SFCT was defined as the vertical distance between the hyperreflective line of the Bruch’s membrane to the hyperreflective line of the inner surface of the sclera. The measurements were performed using the Heidelberg Eye Explorer software (v. 5.3.3.0; Heidelberg Engineering Co, Heidelberg, Germany) (Figure 1). Only the right eye of each study participant was assessed. The interobserver agreement between two ophthalmologists in measuring the SFCT had been assessed in a previous study and had shown correlation coefficient of *r*^2^ = 0.98 (Shao et al., 2013).

We split the dataset into a development dataset and a validation dataset. The division was performed randomly with a ratio of 9:1 for the development/validation dataset. The development dataset consisted of a training set and a tuning set with the proportion of 8:1 (Table 1).

**TABLE 1:** Baseline characteristics (mean ± standard deviation) of participants in the development group and validation group.

	Development set	Validation set	*P* value
Axial length (mm)	23.24 ± 1.15	23.29 ± 1.17	0.49
Number of participants	2,811	313	–
Number of images	5,688	616	–
< 22 mm	506 (8.9%)	55 (8.9%)	–
≥ 22 mm and < 26 mm	5004 (88.0)	546 (88.6%)	–
≥26 mm	178 (3.1%)	15 (2.4%)	–
SFCT (μm)	258.13 ± 106.46	247.65 ± 105.55	0.16
Number of participants	2,672	300	–
Number of images	5,436	592	–
< 150 μm	887 (16.3%)	119 (20.1%)	–
≥ 150 μm and < 350 μm	3498 (64.3%)	364 (61.5%)	–
≥350 μm	1051 (19.3%)	109 (18.4%)	–

*SFCT, subfoveal choroidal thickness.*

For the development of the algorithm, we used a convolutional neural network (CNN), a specialized deep-learning model (Krizhevsky et al., 2012), to analyze the digitized fundus images. The models employed the same configurations and CNN architecture as Inception-Resnet-v2 (Szegedy et al., 2016). Based on this architecture, a modified 164-layer CNN was employed to estimate axial length and SFCT. We initialized the parameters of the neural network with the ImageNet classification pretrained model.

**FIGURE 2:**
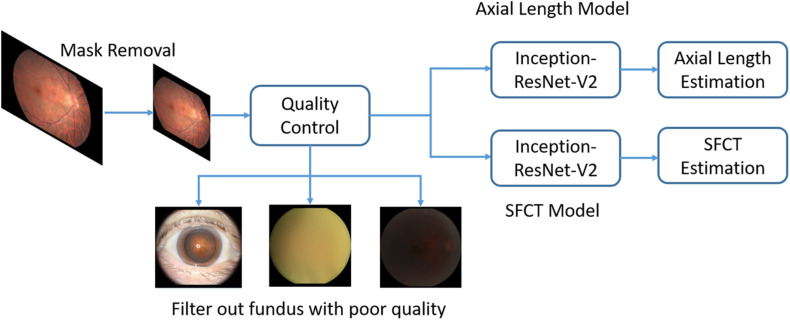
Overview of a deep convolutional neural network (CNN)-based model training pipeline to automatically estimate axial length and subfoveal choroidal thickness from color fundus images.

Before the analysis, we preprocessed the images to improve the CNN-based analysis. We removed the dark background by detecting a circular mask of the photographs, and the images were resized to the size of 500 × 500 pixels. A quality control module was implemented after the mask removal to assess the image quality and to filter out unqualified images (Figure 2). The standard for excluding poor quality images followed the procedures used in previous investigations (Zago et al., 2018) and utilized parameters such as the readable region ratio, illumination, blurriness, and image contents. The pixel values of the selected images applied to a linear mapping with a pixel value ranging from (0, 255) to (0, 1). In the training stage, a batch of images, called the training batch, was generated and fed back to the network. The Huber loss was calculated based on this batch (Huber, 2004). The corresponding gradients of the loss were back-propagated to update the network parameters. We set the batch size (also known as mini-batch size) as 14. The stochastic gradient descent was used for the mini-batch optimization with the learning rate of 0.0001.

To implement and deploy the network, an open-source software library (Keras, V2.2.2^1^) was used for training and evaluation. The model was trained on a dual-GPU of NVIDIA Titan-X with CUDA version 9.0 and cuDNN 7.0. The Inception-ResNet-V2 network architecture used in this work was publicly available in the Keras-Application package.

Since axial length and SFCT are continuous values, the metrics used for the assessment of the model performance were the mean absolute error and the coefficient of determination (*r*^2^). We calculated the mean absolute error and *r*^2^ with their 95% confidence intervals (CIs) with an evaluation of 2000 times. Bland–Altman plotting was used to visualize the agreement between the estimated values and the measured values.

To illustrate the fundus region predominantly used by the CNN to generate and apply the algorithm, we implanted another convolutional visualization layer into our network architecture (Zhou et al., 2016). The layer takes image features learned by the preceding layers and gives each feature a weight indicating its importance. It is shown in heat maps.

## Results

Out of the 3468 participants of the Beijing Eye Study, fundus images of 3124 (90.1%) individuals were eventually included into the present study, after the images of 344 (9.9%) individuals had been excluded due to the exclusion criteria detailed above. Among the included photographs, 3239 images were centered on the macula, and 3065 images were centered on the optic nerve head. For the estimation of axial length, the development group used 5688 retinal fundus images from 2811 participants, and the validation group consisted of 616 images from 313 participants. Since some participants had not undergone OCT imaging, the development group for the estimation of SFCT used 5436 fundus images of the macula from 2672 participants and validated the model using 592 images from 300 participants (Table 1). The mean axial length was 23.24 mm (median, 23.12 mm; range, 18.96–30.88 mm), and the mean SFCT was 257 μm (median, 252 μm; range, 12–854 μm). An axial length between 22 mm and 26 mm was measured for 5550 (88.0%) images, and a SFCT between 150 μm and 350 μm was determined for 3862 (64.1%) images. The development group and the validation group did not differ significantly in axial length (*P* = 0.488) and SFCT (*P* = 0.163).

**FIGURE 3:**
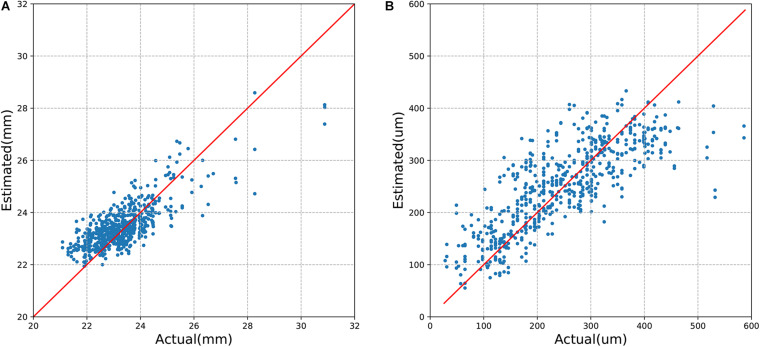
Model performance of estimating (**A**) axial length and (**B**) subfoveal choroidal thickness.

**FIGURE 4:**
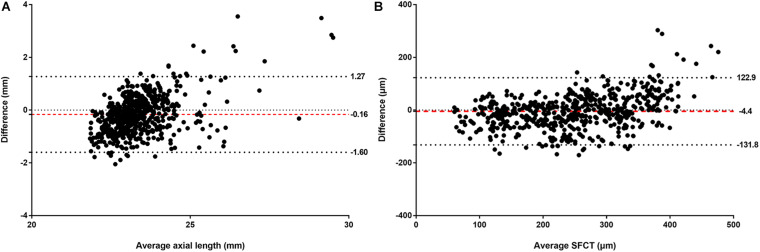
Bland–Altman plots comparing the (**A**) actual and estimated axial length and (**B**) subfoveal choroidal thickness (SFCT). *X*-axis: mean of axial length or SFCT. *Y*-axis: measured values minus the estimated values. The mean differences and the 95% confidence limits of the difference are shown by the three dotted lines.

The mean absolute error (MAE) of the algorithm for the estimation of axial length and SFCT was 0.56 mm (95% CI, 0.53–0.61) and 49.20 μm (95% CI, 45.83–52.54), respectively, with coefficients of determination values of *r*^2^ of 0.59 (95% CI, 0.50–0.65) for axial length and *r*^2^ of 0.62 (95% CI, 0.57–0.67) for SFCT (Table 2). The estimated values and the measured values showed a relatively linear relationship for both parameters (Figure 3). In Bland–Altman plots, the mean difference of axial length was −0.16 mm (95% CI, −1.60–1.27 mm), with 3.7% (23/616) measurement points located outside the 95% limits of agreement (Figure 4). The mean difference of SFCT was −4.40 μm (95% CI, −131.8–122.9 μm), and 4.9% (29/592) of the measurement points were located outside the 95% limits of agreement in the Bland–Altman plots. Subgroup analysis showed the MAE of the algorithm for the estimation of axial length ranged from 22 to 26 mm was 0.50 mm (95% CI, 0.47–0.53), and the MAE for the estimation of SFCT was 42.47 μm (95% CI, 38.80–46.32).

**TABLE 2:** Algorithm performance in the validation set.

Parameters	Performance (95% CI)
**Axial length (*n* = 616)**	
MAE (95% CI), mm	0.56 (0.53, 0.61)
*r*^2^ (95% CI)	0.59 (0.50, 0.65)
**SFCT (*n* = 592)**	
MAE (95% CI), μm	49.20 (45.83, 52.54)
*r*^2^ (95% CI)	0.62 (0.57, 0.67)

*MAE, mean absolute error; CI, confidence interval; SFCT, subfoveal choroidal thickness.*

**FIGURE 5:**
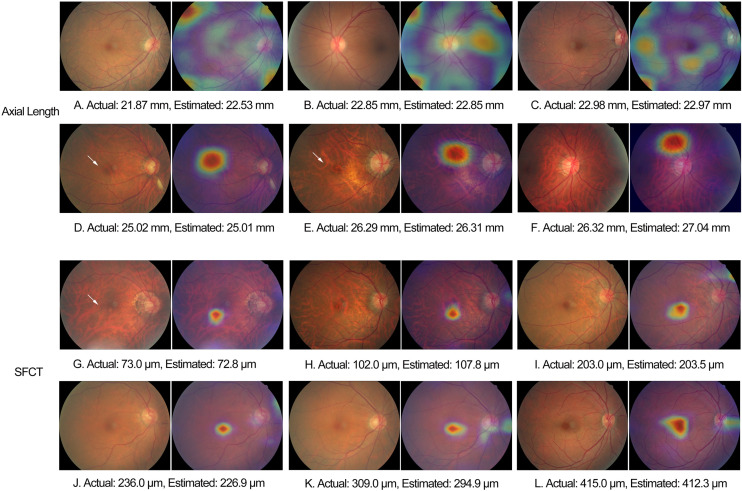
Examples of heat maps generated in eyes of different axial length and subfoveal choroidal thickness. White arrow: fundus tessellation.

For the estimation of axial length, the heat map analysis showed that signals from overall of the macular region were used by the CNN in the eyes with an axial length of < 22 mm, while in the eyes with an axial length ranging between 22 mm and < 26 mm, the CNN used signals mostly from the foveal region, and in the eyes with an axial length of > 26 mm, the CNN used signals from the extrafoveal region within the macular (Figures 5A–F). For the estimation of SFCT, the CNN used mostly the central part of the macular region, the fovea or perifovea, independently of the SFCT (Figures 5G–L).

## Discussion

In our population-based study, the CNN-based algorithm had a mean absolute error for estimating axial length and SFCT of 0.56 mm and 49.20 μm, respectively, and the Bland–Altman plots revealed a mean difference in axial length and SFCT of −0.16 mm and −4.40 μm, respectively.

These results of our study with respect to the estimation of axial length cannot directly be compared with the results of other investigations, since axial length has not been included in a study on deep learning yet. Komuku et al. (2020) used an adaptive binarization method to analyze choroidal vessels on color fundus photographs and a deep-learning-based method to estimate the SFCT based on the binarization-generated choroidal vessel images. The correlations between choroidal vasculature appearance index and choroidal thickness were −0.60 for normal eyes (*P* < 0.01) and −0.46 for eyes with central serous chorioretinopathy (CSC) (*P* < 0.01), respectively. For the deep-learning system, the correlation coefficients between the value estimated from the color images and the true choroidal thickness were 0.68 for normal eyes (*P* < 0.01) and 0.48 for the eyes with CSC (*P* < 0.01), respectively. These values are comparable with the value of *r*^2^ = 0.62 found in our study with a larger study population and a population-based recruitment.

The difference between the estimated values and measured values of the axial length measurements was lower than that between axial length measurements by optical low-coherence reflectometry and sonographic axial length determinations [mean difference, −0.72 mm (95% CI, −0.75, −0.69 mm)] (Gursoy et al., 2011). In that context, it has to be taken into account that it is not the mean difference but the scattering of the difference between two methods that markedly influence the clinical reliability and validity of a technique. The algorithm in our study overestimated axial length for the eyes with a small axial length, and the model underestimated the SFCT in the eyes with a thick SFCT. The findings may be related to an underrepresentation of eyes with a small axial length and eyes with a thick SFCT in the study population. Most eyes included into the study had an axial length ranging between 22 and 26 mm and a SFCT ranging between 150 μm and 350 μm. The advantage of our study population being recruited in a population-based level was combined with the disadvantage of a relative lack of eyes in the extreme range of measurements of axial length and SFCT. Future studies may include preferably such eyes to further improve the algorithm.

The observations made in our study agree with the findings made in other investigations and with clinical experience that axially elongated eyes differ in the appearance of their posterior fundus from the eyes with a short axial length. In a parallel manner, it holds true for the SFCT, since it is strongly correlated with axial length (Fujiwara et al., 2009; Wei et al., 2013). A main feature of an axially elongated eye is an increased degree of fundus tessellation, which is also strongly correlated with a decreasing thickness of the SFCT (Yan et al., 2015). Other features of an increasing axial elongation in non-highly myopic eyes include a shift of the Bruch’s membrane (BM) opening, usually into the temporal direction, leading to an overhanging of BM into the intrapapillary compartment at the nasal optic disk and, correspondingly, an absence of BM at the temporal disk border in the form of a parapapillary gamma zone; an ovalization of the ophthalmoscopically detectable optic disk shape and a decrease in the ophthalmoscopical horizontal disk diameter due to the temporal BM shift; and an increase in the disk–fovea distance due to the development of parapapillary gamma zone and, correspondingly, a decrease in the angle kappa between the two temporal vascular arcades (Jonas et al., 2015, 2017, 2019; Guo et al., 2018). In view of this long list of axial elongation-associated morphological changes in the posterior fundus, it might have been expected that besides ophthalmologists, also deep-learning-based algorithms can estimate axial length. Interestingly, the heat map analysis revealed that signals predominantly from overall of the macular region, the foveal region, and the extrafoveal region were used in eyes with an axial length of < 22 mm, 22–26 mm, and > 26 mm, respectively. For the estimation of SFCT, the CNN used mostly the central part of the macular region, the fovea or perifovea, independently of the SFCT. It agrees with the finding of a previous study that the degree of fundus tessellation assessed in the macular region or in parapapillary region can be used to estimate SFCT and that a high degree of fundus tessellation is a surrogate for a leptochoroid (Yan et al., 2015).

The practical importance of an algorithm estimating the axial length may be in a combination of portable and cheap fundus cameras with such an algorithm (Bastawrous et al., 2016; Toy et al., 2016; Muiesan et al., 2017; Mamtora et al., 2018). Based on the data available so far, it may be unlikely that a deep-learning algorithm based only on fundus photographs will be better than biometry for the measurement of axial length. The same may hold true for the assessment of SFCT.

When the results of our study are discussed, its limitations should be taken into account. First, the study population included only subjects aged ≥ 50 years, so the results of our study cannot directly be transferred to younger individuals. Second, by the same token, the study population consisted only of Chinese so that future studies may address study population of different ethnicity. Third, the use of both optic-disk-centered fundus images and macula-centered fundus photographs, for the training and validation of the algorithm, might have led to some scattering in estimations. However, it should be noticed that the fovea was visible also on the optic nerve head images, and vice versa, the optic disk was visible on the macula-centered photographs. It indicates that the fovea, as the most important part for the estimation of the SFCT and axial length, was assessable in both types of photographs. In addition, the optic nerve head shows characteristic of axial-length-related particularities, so that the inclusion of its full image in the optic-disk-centered images might only have supported finding a best fitting algorithm. It also holds true for the estimation of the SFCT since the SFCT is strongly correlated with axial length (Liu et al., 2018). Adding the optic nerve head photographs to the study, furthermore, increased the sample size for the training of the model. Fourth, the attention maps did not rule out that other features in the images were also used, and we did not perform a quantitative validation of the heat maps. Fifth, although the study population as a real-world group also included eyes with disorders of the macula and optic nerve, we did not analyze whether the inclusion of eye with disorders influenced the performance of the algorithm. Sixth, we did not include a second data set of a completely different study population so that the validation of the algorithm can still be further refined. Further research may include data sets from populations of different age ranges and ethnicities and may use different fundus cameras. In addition, to boost the performance of the model, one may use more data for the development of the algorithm and improve the training schemes, such as using data augmentation.

In conclusion, deep-learning-based algorithms may be helpful for estimating axial length and SFCT based on conventional color fundus images. They may be a further step in the semiautomatic assessment of the eye.

## Data Availability Statement

The raw data supporting the conclusions of this article will be made available by the authors, without undue reservation.

## Ethics Statement

The studies involving human participants were reviewed and approved by the Ethics Committee of Beijing Tongren Hospital. The patients/participants provided their written informed consent to participate in this study. Written informed consent was obtained from the individual(s) for the publication of any potentially identifiable images or data included in this article.

## Author Contributions

LD, YNY, QZ, NZ, YXW, and WBW: design of the study. XYH, JHX, CXL, and ZYG: development of the algorithm. YNY, QZ, YXW, JX, LS, YJL, and YL: gathering the data. LD, XYH, YNY, QZ, NZ, YXW, JX, and JBJ: performing the data analysis. LD, XYH, and JHX: drafting the first version of the manuscript. All authors: revision and approval of the manuscript.

## Conflict of Interest

XYH, JHX, and CXL were employed by the company Beijing Eaglevision Technology Co., Ltd., China The remaining authors declare that the research was conducted in the absence of any commercial or financial relationships that could be construed as a potential conflict of interest

## Footnotes

1 https://github.com/fchollet/keras
